# Assessing the Predictive Value of Methicillin-Resistant *Staphylococcus aureus* Nares Colonization Among Transplant Recipients and Patients With Neutropenia

**DOI:** 10.1093/ofid/ofae408

**Published:** 2024-07-16

**Authors:** Reid Shaw, Alison Zander, Tanisha Ronnie, Zubeen Azari, Alex Gregorowicz, Fritzie Albarillo

**Affiliations:** Department of Internal Medicine, Loyola University Medical Center, Maywood, Illinois, USA; Department of Internal Medicine, Loyola University Medical Center, Maywood, Illinois, USA; Department of Internal Medicine, Loyola University Medical Center, Maywood, Illinois, USA; Department of Internal Medicine, Loyola University Medical Center, Maywood, Illinois, USA; Department of Pharmacy, Edward Hines Veterans Affairs Hospital, Hines, Illinois, USA; Division of Infectious Disease, Loyola University Medical Center, Maywood, Illinois, USA

**Keywords:** Antibiotic stewardship, Methicillin-resistant *Staphylococcus aureus*, MRSA nares, neutropenia, transplant recipients

## Abstract

**Background:**

Methicillin-resistant *Staphylococcus aureus* (MRSA) nares screening has been shown to be a powerful antibiotic stewardship tool for MRSA infections within 7 days of screening across a variety of anatomical locations given the high negative predictive value (NPV). However, the utility outside of 7 days and among transplant recipients and patients with neutropenia is less clear.

**Methods:**

This was a retrospective cohort study across Veterans Affairs medical centers in the United States from 1 January 2007 to 1 January 2023 of patients tested for MRSA colonization and who had a subsequent positive bacterial culture within 28 days of MRSA sc­­­reening. Sensitivity, specificity, positive predictive value, and NPV were calculated across different time points and anatomical culture locations.

**Results:**

The cohort consisted of 686 174 patients, 6 277 437 MRSA nares tests, and 2 446 766 positive bacterial cultures within 28 days of MRSA testing. The NPV of MRSA nares screening for ruling out a MRSA infection within 28 days was 95.8% across all anatomical culture sites. The NPV was 97.9% among patients with neutropenia and 97.5% in solid organ and hemopoietic stem cell transplant recipients.

**Conclusions:**

MRSA nares screening can reliably be used for de-escalation of anti-MRSA therapy within 28 days of bacterial culture for all patients, including solid organ and hematopoietic transplant recipients and patients with neutropenia.

Methicillin-resistant *Staphylococcus aureus* (MRSA) nares polymerase chain reaction (PCR) screening has proven to have a high negative predictive value (NPV) for MRSA infections across a variety of anatomical sites within 7 days of microbiological culture [1, 2]. This has allowed for the confident de-escalation and avoidance of empiric anti-MRSA treatment before the finalization of cultures [3, 4]. However, MRSA nares screening is not well validated in high-risk individuals, including those with neutropenia, hematopoietic stem cell transplant (HSCT) recipients, and solid organ transplant (SOT) recipients [5, 6].

Febrile neutropenia is a common and potentially life-threatening condition in cancer patients, with mortality rates approaching 10% [7]. Infectious Diseases Society of America (IDSA) guidelines recommend empiric anti–*Pseudomonas aeruginosa* (*Pa*) coverage for all patients [8]. In patients with a suspected catheter-related skin or soft tissue infection, pneumonia, or hemodynamic instability, the National Comprehensive Cancer Network and IDSA recommend adding anti-MRSA treatment [8, 9]. In SOT recipients, the rates of opportunistic infections are high due to the immunosuppressive medications required to prevent graft rejection [10]. MRSA infections in SOT recipients are associated with longer hospital stays, increased hospital costs, and higher rates of mortality [11, 12]. However, the overuse of vancomycin is associated with increased adverse events and hospital costs [13]. Therefore, timely and precise microbiological diagnosis is crucial to direct treatment and reduce unnecessary drug therapy [14]. The primary goal of this study is to assess the predictive value of MRSA nares among high-risk individuals, including patients with neutropenia, HSCT recipients, and SOT recipients.

Risk factors for multidrug-resistant organisms (MDROs) are similar; thus, empiric broad-spectrum antibiotics constitute the backbone of therapy in critically ill patients [15–17]. However, the IDSA guidelines recommend de-escalation of empiric therapy at 48 hours if microbial cultures do not identify an MDRO and the patient is clinically improving [18]. While de-escalation aims to reduce antibiotic-related toxicity and limit the development of MDRO, this process is often delayed [19]. Thus, the second goal of this study is to assess the predictive value of MRSA nares colonization for MDRO.

## METHODS

A multicenter retrospective cohort study was conducted among the Veterans Affairs (VA) medical system from 1 January 2007 to 1 January 2023. All VA locations were queried, but results for Iowa and Nebraska are combined and Kansas is combined with Missouri due to overlapping station coding. Patients aged ≥18 years were queried for MRSA nares testing within the VA's Corporate Data Warehouse (CDW), which stores all VA patient medical data. MRSA colonization was assessed with PCR and standard culture techniques. Data were queried from CDW using structured query language (SQL) via the SQL Server Management Studio, and statistical analysis was completed with R programming language v.4.3.1 [20]. The VA Informatics and Computing Infrastructure (VINCI)—a virtual machine architecture with dynamic memory—was used. Acquisition and analysis of the data was approved by the Edward Hines VA Institutional Review Board.

Date of birth, sex, race, hospital admission dates, absolute neutrophil count, *International Classification of Diseases* codes, and positive microbiological cultures within 28 days of MRSA colonization assessment were collected for all patients. The queried microbiological cultures of interest included MRSA, vancomycin-resistant *Enterococcus*, carbapenem-resistant Enterobacteriaceae, extended-spectrum β-lactamase (ESBL)–producing gram-negative bacteria, *Pa*, *Burkholderia*, *Acinetobacter*, and *Stenotrophomonas.*

Neutropenia was defined as an absolute neutrophil count of <1500 cells/µL. Transplants included hematopoietic stem cells and solid organs, including the pancreas, lungs, liver, kidney, intestine, and heart. Culture sites were grouped by the anatomical collection site: wound, urinary, blood, intra-abdominal, pulmonary, reproductive, central nervous system, graft, device, drain, stool, and others. MRSA nares colonization was assessed using culture and PCR, but PCR results were only used to calculate the predictive performance given the rapid turnaround time and high accuracy. In the case of multiple MRSA tests being performed within 28 days of a positive culture, pseudo-random sampling was performed to eliminate bias from clustered data [21]. Sensitivity, specificity, positive predictive value (PPV), and NPV were calculated. Proportional testing assessed for differences among proportions [22]. Local polynomial regression fitting tested individual culture trends over time [23].

## RESULTS

**Figure 1.**
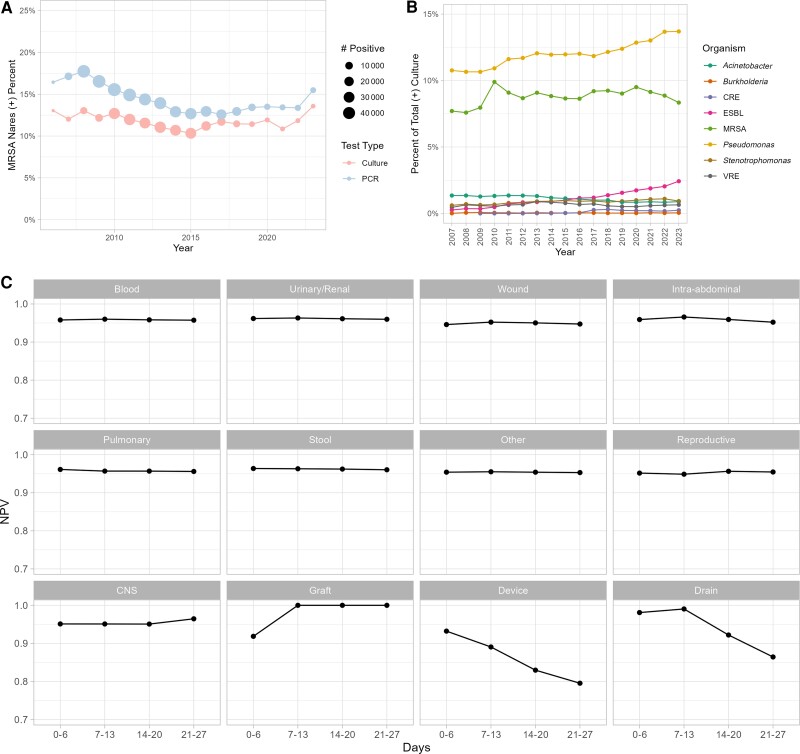
*A*, Incidence of positive methicillin-resistant *Staphylococcus aureus* (MRSA) nares colonization, stratified by test type. *B*, Incidence of multidrug-resistant organisms across the study period. *C*, Negative predictive value of MRSA infections. Abbreviations: CNS, central nervous system; CRE, carbapenem-resistant *Enterococcus*; ESBL, extended-spectrum β-lactamase; MRSA, methicillin-resistant *Staphylococcus aureus*; NPV, negative predictive value; VRE, vancomycin-resistant *Enterococcus*.

A total of 686 174 patients were screened for MRSA nares colonization, generating 6 277 437 total tests. The patient population was predominantly White (72.2%) and male (95.5%) with a median age of 68.7 years (Table 1). Of the MRSA nares screening tests, 59.1% were tested with PCR and 40.9% with standard culture. The percent positive of PCR and standard culture was 14.3% and 11.4%, respectively (*P* < .001; Figure 1*A*).

**Table 1.** Patient Demographics (N = 558 675)

Characteristic	No. (%)
Age, y, mean ± SD	68.7 ± 12
Sex	
Male	533 727 (95.5)
Female	24 948 (4.5)
Race	
White	403 940 (72.3)
Black or African American	111 158 (19.9)
Declined to answer	21 031 (3.7)
Unknown	10 461 (1.9)
Native Hawaiian or Pacific Islander	4961 (0.9)
American Indian or Alaska Native	4916 (0.9)
Asian	2208 (0.4)
Test	
Culture	2 569 454 (40.9)
PCR	3 707 983 (59.1)

Abbreviations: PCR, polymerase chain reaction; SD, standard deviation.

There were 2 446 766 positive culture results identified within 28 days of MRSA testing. The percent positive rate of MRSA colonization was 23.1% in individuals with a subsequent positive culture. *Pa* was the most common organism within our cohort at 10.6% while MRSA was second at 8.5% (Figure 1*B*). No other MDROs had an incidence of >2.5% in any of the study years. The incidence of ESBL-producing organisms, MRSA, and *Pa* increased throughout the study period (Figure 1*B*). *Burkholderia* and carbapenem-resistant *Enterococcus* had the lowest incidence among MDRO at 0.04% and 0.09%, respectively. Bacterial culture prevalence was stratified by state (Supplementary Table 1). The prevalence of MRSA infections was lowest in Maryland at 0.06% and highest in Alaska at 14.7%. The prevalence of *Pa* infections was lowest in Vermont at 6.5% and highest in Alaska at 20.7%.

MRSA nares screening provided for a high NPV among all MDROs (Table 2). Across all anatomical culture sites, the NPV of MRSA infections was 95.8% within 28 days of MRSA nares screening (Table 2). The NPV for devices and drains trended lower the farther screening was done from culture collection (Figure 1*C*). The NPV across all other anatomical sites remained consistent across the 28 days (Figure 1*C*). Interstate variability of MRSA nares predictive value was assessed for MRSA infections. The NPV was >91.8% for all states except for Montana, which had an NPV of 87.1% (Supplementary Table 2). The NPV for *Pa* decreased the most across the study period, starting at 88.8% and falling to 84.6% (Supplementary Figure 1).

**Table 2.** Summary Statistics of Methicillin-Resistant *Staphylococcus aureus* Nares Testing Within 4 Weeks of Positive Bacterial Culture for Multidrug-Resistant Organisms

Organism	Sensitivity, %	Specificity, %	PPV, %	NPV, %
*Burkholderia*	12.2	76.9	0.03	99.9
CRE	26.5	76.9	0.15	99.9
*Acinetobacter*	27.4	77.0	1.44	98.9
ESBL	25.7	76.9	1.32	98.9
VRE	21.0	76.9	1.00	98.9
*Stenotrophomonas*	16.2	76.8	0.83	98.7
MRSA	66.6	81.6	27.8	95.8
*Pseudomonas*	22.4	76.8	12.7	86.8

Abbreviations: CRE, carbapenem-resistant *Enterococcus*; ESBL, extended-spectrum β-lactamase–producing organism; MRSA, methicillin-resistant *Staphylococcus aureus*; NPV, negative predictive value; PPV, positive predictive value; VRE, vancomycin-resistant *Enterococcus*.

There were 2149 patients with neutropenia; of those, 761 (35.4%) were patients with a hematologic malignancy. Among the patients with neutropenia, there were 3212 positive bacterial cultures identified within 4 weeks of MRSA nares screening. Of the positive cultures, there were 188 (5.9%) MRSA infections. The incidence of MRSA and *Pa* infections was significantly lower in the neutropenic cohort compared to the entire cohort (*P* < .001) (Supplementary Figure 2). Vancomycin-resistant *Enterococcus* (VRE) infections were significantly more common in patients with neutropenia compared to the complete cohort (*P* < .001; Supplementary Figure 2). The NPV of MRSA nares colonization was 97.9% for MRSA infections across all anatomical locations and >88% across all MDROs (Table 3).

**Table 3.** Neutropenic Patient Summary Statistics of Methicillin-Resistant *Staphylococcus aureus* Nares Testing Within 4 Weeks of a Positive Bacterial Culture

Organism	Sensitivity, %	Specificity, %	PPV, %	NPV, %
*Burkholderia*	0	84.9	0	99.9
CRE	0	84.9	0	99.9
*Acinetobacter*	24.6	85.0	1.6	99.9
*Stenotrophomonas*	5.0	85.0	0.4	98.6
ESBL	21.8	85.0	2.5	98.4
MRSA	72.4	88.9	30.9	97.9
VRE	10.3	84.7	3.6	94.4
*Pseudomonas*	11.7	84.5	8.5	88.6

Abbreviations: CRE, carbapenem-resistant *Enterococcus*; ESBL, extended-spectrum β-lactamase–producing organism; MRSA, methicillin-resistant *Staphylococcus aureus*; NPV, negative predictive value; PPV, positive predictive value; VRE, vancomycin-resistant *Enterococcus*.

Among the entire cohort, 19 909 patients received an SOT or HSCT with 1658 patients receiving >1 transplant. There were 9294 kidney transplant recipients, 5226 liver transplant recipients, 3761 HSCT recipients, 2101 heart transplant recipients, 1051 lung transplant recipients, 233 pancreas transplant recipients, and 29 intestine transplant recipients. Among transplant recipients, the incidence of *Pa* infections was highest at 13.6%, MRSA at 6.8%, VRE at 2.8%, ESBL-producing organisms at 1.9%, *Stenotrophomonas* at 1.6%, *Acinetobacter* at 0.1%, and *Burkholderia* at 0.04%. The incidence of *Pa* and ESBL infections increased over the study period (Supplementary Figure 3). For MRSA infections, the NPV was 97.5% among HSCT and SOT recipients within 28 days of MRSA nares testing (Table 4). The cumulative NPV for *Pa* was 85.7% (Table 4). However, the NPV was lowest in lung transplant recipients at 71.1% (Supplementary Figure 4).

**Table 4.** Transplant Recipient Summary Statistics of Methicillin-Resistant *Staphylococcus aureus* Nares Testing Within 4 Weeks of Positive Bacterial Culture for Multidrug-Resistant Organisms

Organism	Sensitivity, %	Specificity, %	PPV, %	NPV, %
*Burkholderia*	0	83.6	0	99.9
CRE	18.3	83.6	0.2	99.9
*Acinetobacter*	12.7	83.6	0.5	99.4
*Stenotrophomonas*	16.5	83.6	1.6	98.4
ESBL	14.3	83.6	1.5	98.2
MRSA	71.6	87.9	31.8	97.5
VRE	5.9	83.3	0.9	97.2
*Pseudomonas*	11.7	82.9	9.6	85.7

Abbreviations: CRE, carbapenem-resistant *Enterococcus*; ESBL, extended-spectrum β-lactamase–producing organism; MRSA, methicillin-resistant *Staphylococcus aureus*; NPV, negative predictive value; PPV, positive predictive value; VRE, vancomycin-resistant *Enterococcus*.

## DISCUSSION

Although early active antibiotic therapy is associated with improved outcomes in patients with sepsis, appropriate and timely de-escalation is also important [24]. Anti-MRSA therapy is associated with a higher risk of kidney injury, *Clostridioides difficile* infection, VRE infection, and secondary gram-negative rod identification [25]. Unnecessarily broad antimicrobial treatment is also associated with higher mortality rates [26]. Recently, MRSA nares screening has been shown to be a valuable tool in the de-escalation of anti-MRSA therapy, demonstrating a high predictive value for MRSA infections across all anatomical locations within 7 days of screening [1, 2]. In this retrospective cohort study, we assessed an expanded time from MRSA colonization testing to microbial culture, demonstrating a high NPV within 28 days of colonization testing. While smaller studies have reported a high NPV for respiratory cultures outside of the 7-day window, we assessed all anatomical culture locations [27]. We found that for the majority of anatomical locations, the NPV remained high. This will allow for the confident avoidance of empiric anti-MRSA coverage, with fewer MRSA nares screening tests throughout a patient's hospitalization, decreasing testing burden and, potentially, healthcare costs.

The literature on high-risk patient populations and MRSA nares screening is limited to pneumonia. Recently, a small study (n = 98) investigated patients with acute myeloid leukemia and pneumonia, demonstrating a high PPV and NPV for MRSA nares screening [5]. However, to our knowledge, no prior work has specifically investigated the predictive value of MRSA nares across other anatomical locations in patients with neutropenia or hematological malignancies. In febrile neutropenia, the most common source of bacteremia is from enteric bacteria translocation into the blood. Gram-negative rods, particularly Enterobacteriaceae, and *Pa* are the most common causative organisms and are associated with high rates of mortality [28]. The IDSA recommends anti-*Pa* coverage with either a fluoroquinolone or a β-lactam agent [8, 29]. The IDSA also recommends against the use of agents targeting aerobic gram-positive cocci unless the suspected source is a catheter-related infection, a skin or soft tissue infection, or pneumonia or the patient demonstrates hemodynamic instability. Although hemodynamic stability was not included in our analysis, MRSA nares screening had a high NPV for catheter-related, skin, soft tissue, and lung infections. The judicious use of vancomycin is particularly important in patients with neutropenia as antibiotic stewardship aims to limit the selection of resistant organisms, including VRE, which we found to be 5 times more prevalent in patients with neutropenia.

SOT and HSCT recipients are 2 additional groups of patients who are at high risk for opportunistic infections, including MRSA infections [14, 30]. To our knowledge, there have been no published data on the predictive value of MRSA nares in these specific high-risk groups. Overall, the NPV remains consistent with other patient groups, highlighting the potential clinical utility for anti-MRSA de-escalation and avoidance. However, the consequences of untreated MRSA infections can be significant and antibiotic de-escalation must be considered closely [31]. In this study, we did not attempt to assess the net benefit or harm that empirical treatment may provide. The overall NPV of this study is likely underestimated as the prevalence of MRSA colonization is higher in this patient cohort compared to the general population due to the older age of study participants [32]. Prospective trials are needed to establish the safety and efficacy of MRSA nares screening to guide antibiotic therapy, especially in high-risk patient populations.

The IDSA recommends empiric MRSA and *Pa* coverage in severe infections. However, there are currently no well-validated PCR-based de-escalation tools for *Pa* [17]. Therefore, anti-*Pa* treatment is guided by standard microbiological cultures. Given the similar risk factors for MRSA and *Pa* infections (ie, recent intravenous antibiotic use, hospital admission), we hypothesized that MRSA nares may be a useful tool for de-escalation of anti-*Pa* therapy [18]. We found an NPV of 86.8% for *Pa* infections. However, the likelihood ratio and the incidence of *Pa* infections preclude the screening tool from any clinical utility. Future work will investigate alternative laboratory tests and clinical data for the effective de-escalation of empiric *Pa* coverage.

## CONCLUSIONS

In the current study, we observed a high NPV of MRSA colonization for MRSA infections across various anatomical locations within 28 days of testing across a large patient cohort across the United States, including transplant recipients and patients with neutropenia.

## Supplementary Material

ofae408_Supplementary_Data

## Supplementary Data

Supplementary materials are available at *Open Forum Infectious Diseases* online. Consisting of data provided by the authors to benefit the reader, the posted materials are not copyedited and are the sole responsibility of the authors, so questions or comments should be addressed to the corresponding author.

## Notes

***Acknowledgments*.** This research is the result of work supported with resources and the use of facilities at the Edward Hines VA.

***Author contributions.*** R. S. and F. A. contributed to study conceptualization. All authors contributed to study design, analysis, and interpretation. R. S. contributed to data collection and processing. R. S. led development of the first draft of the manuscript. All authors revised the paper critically, made substantiative intellectual contributions to the development of the manuscript, and approved the final version.

***Data availability.*** The data are owned by the VA and will not be made public. Further information regarding analytical code is available upon request from the corresponding author.

***Patient consent.*** This research was conducted following the guidelines set forth by the Institutional Review Board (IRB) at Hines VA Medical Center. The study protocol was reviewed and approved by the IRB to ensure ethical standards were met.
