# Supplementary figures and images for: Assessing the Predictive Value of Methicillin-Resistant Staphylococcus aureus Nares Colonization Among Transplant Recipients and Patients With Neutropenia
Source: Open Forum Infect Dis. 2024 Jul 16;11(7):ofae408. doi: 10.1093/ofid/ofae408 (PMC11267222; doi:10.1093/ofid/ofae408)

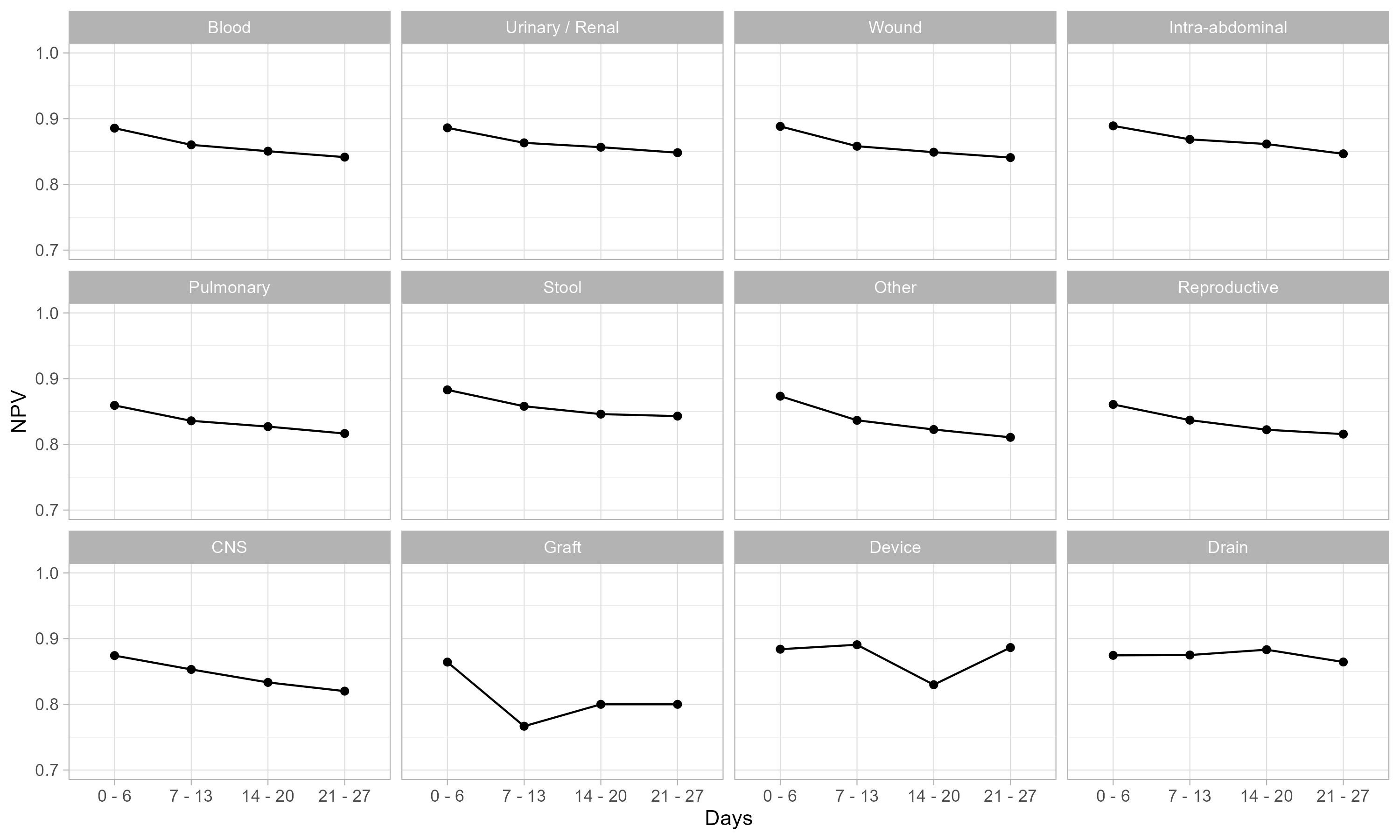

Supplement: ofae408_Supplementary_Data [file ofae408_supplementary_data.zip › Supp_figure_1.tiff]

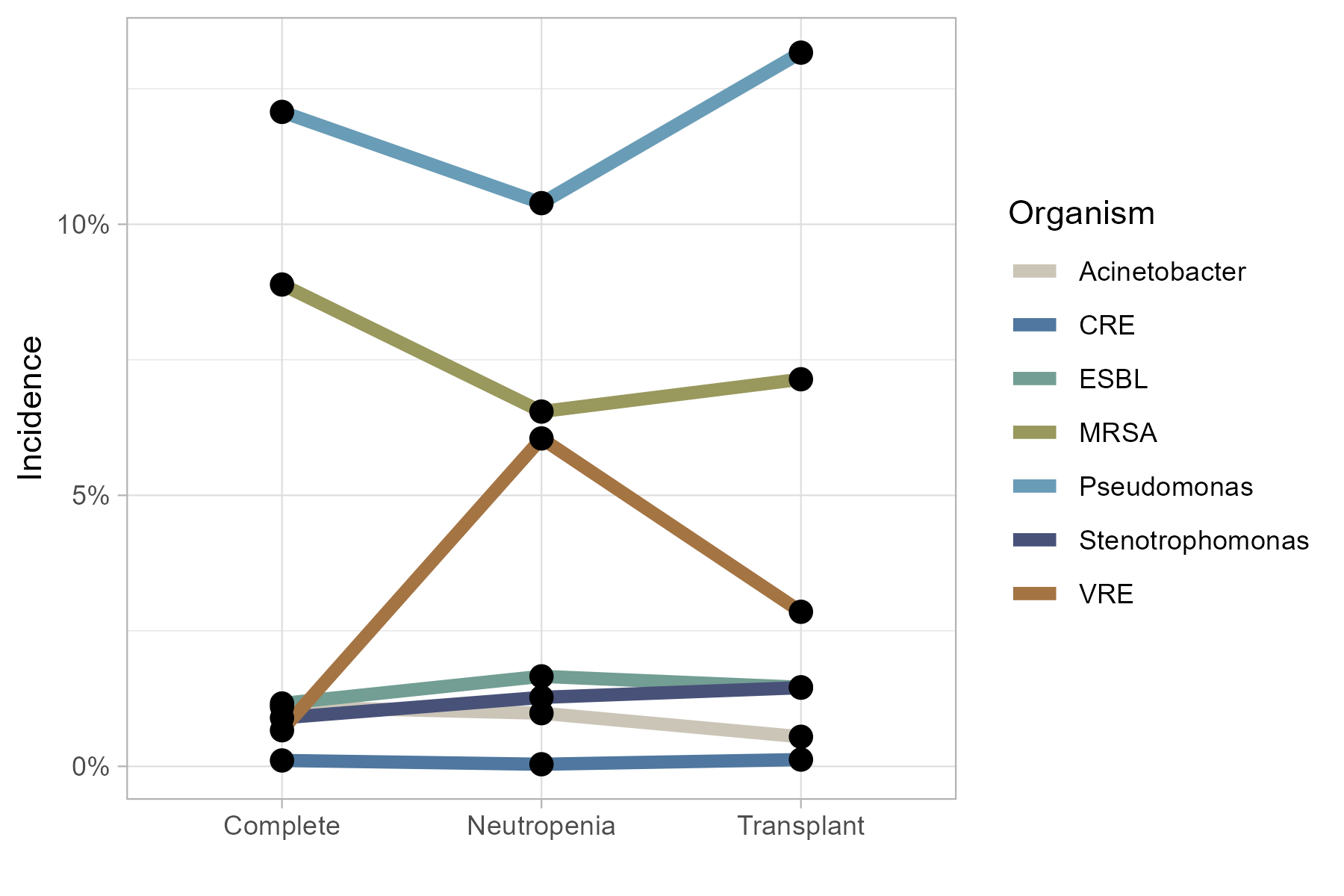

Supplement: ofae408_Supplementary_Data [file ofae408_supplementary_data.zip › Supp_figure_2.tiff]

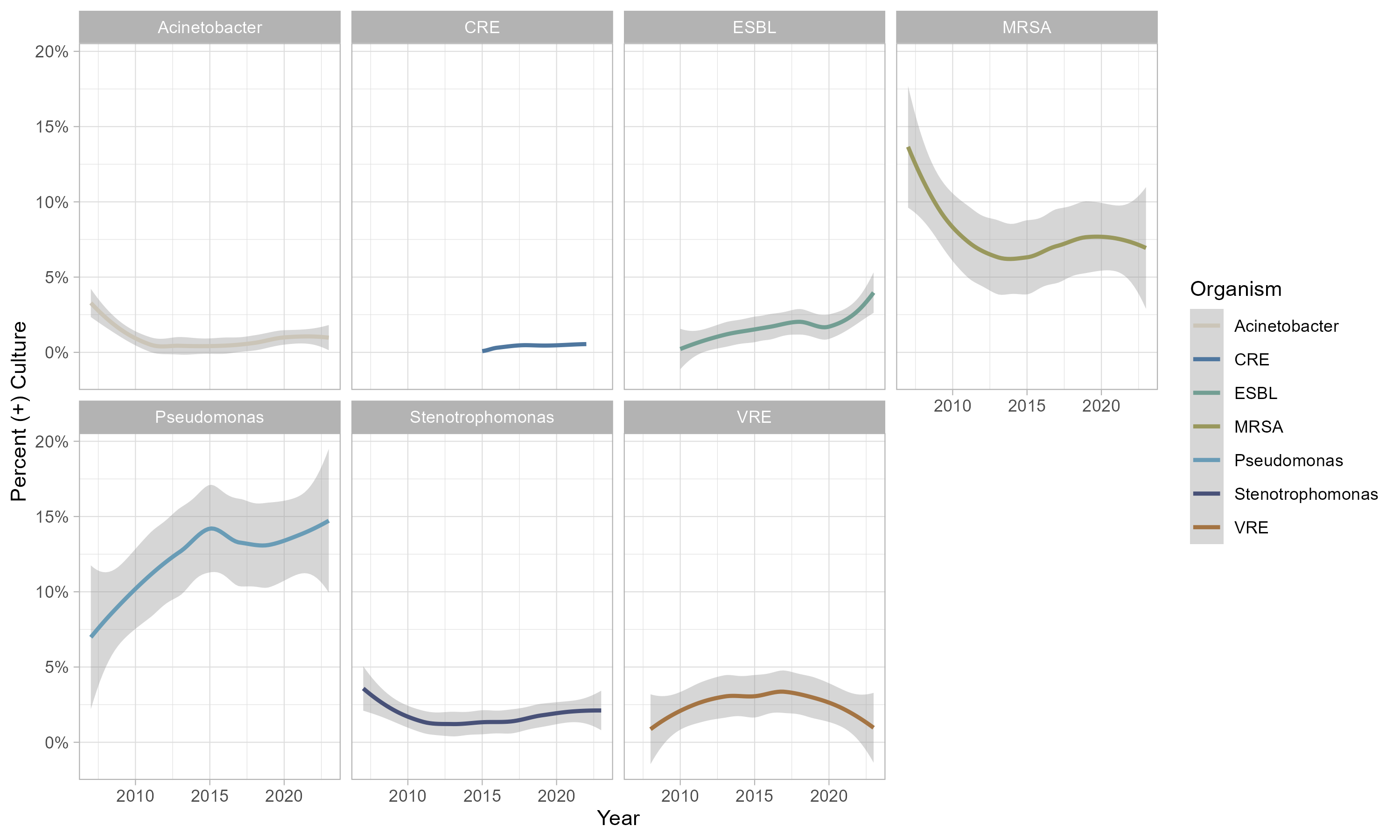

Supplement: ofae408_Supplementary_Data [file ofae408_supplementary_data.zip › Supp_figure_3.tiff]

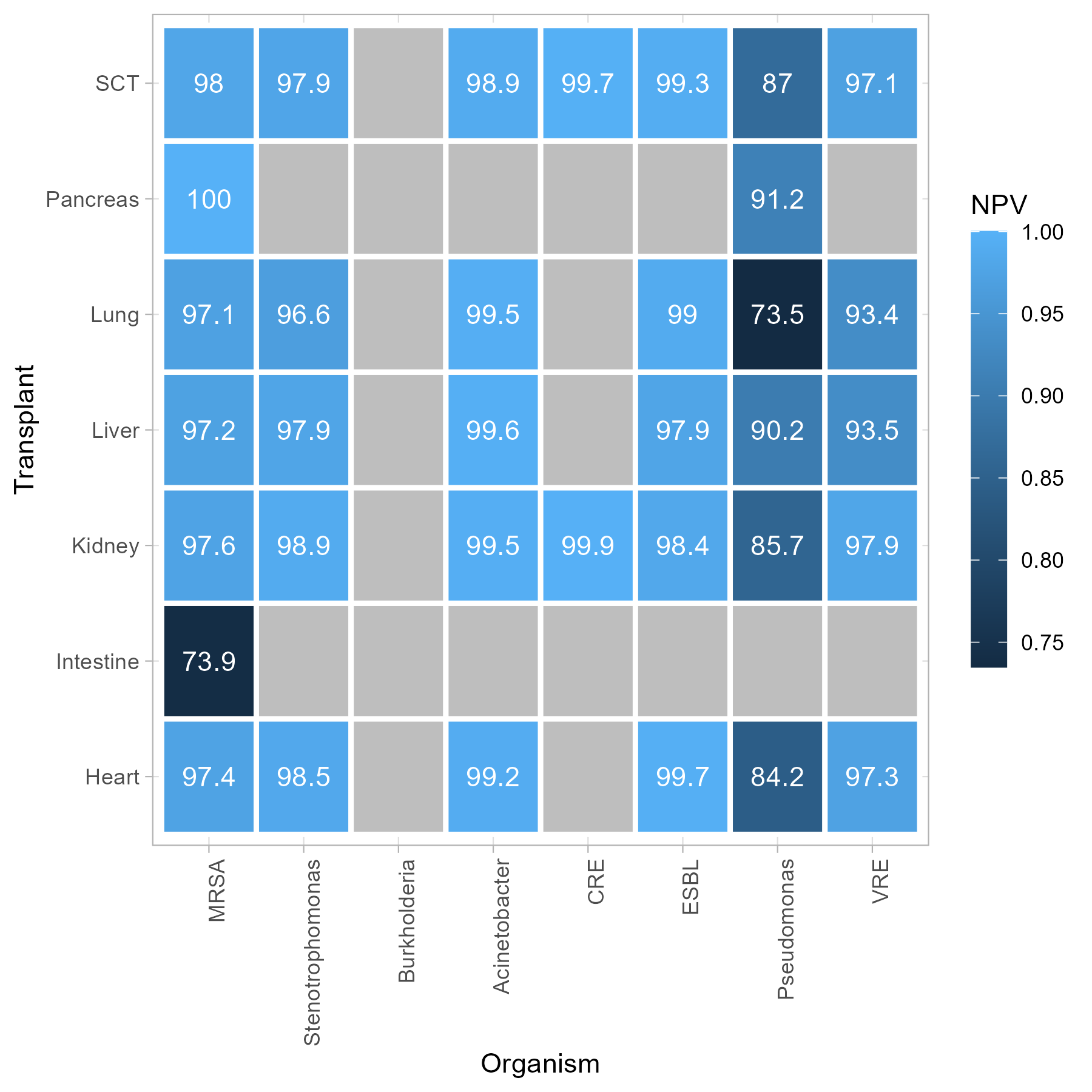

Supplement: ofae408_Supplementary_Data [file ofae408_supplementary_data.zip › Supp_figure_4.tiff]
